# Effects of cognitive and motor dual-tasks on oropharyngeal swallowing assessed with FEES in healthy individuals

**DOI:** 10.1038/s41598-020-77421-3

**Published:** 2020-11-23

**Authors:** Paul Muhle, Inga Claus, Bendix Labeit, Mao Ogawa, Rainer Dziewas, Sonja Suntrup-Krueger, Tobias Warnecke

**Affiliations:** 1 Department of Neurology with Institute for Translational Neurology, University Hospital Muenster, Albert-Schweitzer-Campus 1; Gebäude A1, 48149 Münster, Germany; 2 Institute of Biomagnetism and Biosignalanalysis, University Hospital Muenster, University of Muenster, Malmedyweg 15, 48149 Münster, Germany; 3 Department of Rehabilitation Medicine I, School of Medicine, Fujita Health University, 1-98 Dengakugakubo, Kutsukake, Toyoake, Aichi 470-1192 Japan

**Keywords:** Neurology, Neurophysiology

## Abstract

Dysphagia is frequent in many neurological diseases and gives rise to severe complications such as malnutrition, dehydration and aspiration pneumonia. Therefore, early detection and management of dysphagia is essential and can reduce mortality. This study investigated the effect of cognitive and motor dual-task interference on swallowing in healthy participants, as dual-task effects are reported for other motor tasks such as gait and speech. 27 participants (17 females; 29.2 ± 4.1 years) were included in this prospective study and examined using flexible endoscopic evaluation of swallowing (FEES). Using a previously established FEES-based score, the paradigms “baseline swallowing”, “cognitive dual-task” and “motor dual-task” were assessed. Scores of the three paradigms were compared using a repetitive measures ANOVA and post-hoc analysis. Mean baseline swallowing score in single task was 5 ± 3. It worsened to 6 ± 5 in the cognitive (*p* = 0.118), and to 8 ± 5 in the motor dual-task condition (*p* < 0.001). This change was driven by subclinical worsening of premature bolus spillage and pharyngeal residue. Oropharyngeal swallowing is not exclusively reflexive in nature but requires attention, which leads to motor dual-task interference. This has potential diagnostic and therapeutic implications, e.g. in the early screening for dysphagia or in avoiding dual-task situations while eating.

## Introduction

Oropharyngeal dysphagia frequently occurs in several neurological disorders such as stroke^1^, Parkinson's disease^2^, dementia^3,4^, multiple sclerosis^5^ or neuromuscular disorders such as inflammatory myopathies or myasthenia gravis^6–8^, but also as age-related dysphagia in elderly otherwise healthy people^9^. Dysphagia not only affects patients' quality of life^10^, but is also prone to severe complications such as malnutrition, dehydration and aspiration pneumonia resulting in worse long-term outcome and increased mortality^1,8,11–20^. In fact, pneumonia after aspiration is often a leading cause of death in these patient groups^1,8,18,20–23^. Conversely, mortality can be reduced by systematic dysphagia management, which requires early screening and detection of swallowing impairment^16,24^. Instrumental assessment is necessary to validly evaluate safety and efficiency of deglutition because relevant swallowing sequelae, such as silent aspiration, are likely to be overlooked in clinical examination^25^. In this context, flexible endoscopic evaluation of swallowing (FEES) is considered a feasible and safe diagnostic gold standard in neurogenic dysphagia^26^.

In everyday life, eating often takes place concurrently to other activities such as talking in a group^27^ or watching television^28,29^. Therefore, we wanted to determine the effect of multitasking on swallowing. The standard FEES protocols for neurogenic dysphagia are mainly limited to the examination of volitional swallowing in a more or less artificial situation^30–34^. This examination context reduces ecological validity by drawing the attention of the examined patient exclusively to swallowing. Several previous studies have shown that the simultaneous execution of two tasks (dual-task) often results in delayed reaction time or deteriorated performance in one or both of the tasks^35–37^. This phenomenon is referred to as dual-task cost^35^ and was shown in various motor tasks such as gait^36^ or speech^37^ but also in more autonomous functions like breathing^38^. It not only occurs in healthy subjects but even stronger in patients with neurological disorders such as Parkinson's disease^35,39^. We therefore hypothesized that dual-task interference might also impair the complex neuromuscular processing of swallowing which requires the precise central coordination of multiple muscle pairs via cranial nerves. To evaluate this hypothesis, we investigated the effect of concurrent motor and cognitive dual-tasks on oropharyngeal swallowing using FEES in healthy individuals.

## Results

Ten subjects were male (37%). The mean age was 29.2 ± 4.1 years [range 22–47 years]. The average swallowing score was significantly higher [F(2,52) = 10.91, *p* < 0.001] in the dual-task conditions compared to the single task condition. Also, the subdomains of premature bolus spillage [F(2,52) = 6.03, *p* = 0.004] and pharyngeal residue [F(1.58,40.99) = 10.91, *p* = 0.005] were significantly higher in the dual-task condition whereas penetration/aspiration did not differ significantly. If residues occurred, during motor dual-task they presented mainly in the valleculae (12/27 participants), in one participant in the piriform sinus and in 3 participants in both locations. During the cognitive dual-task in 13 participants residues were detected in the valleculae, in one participant in the piriform sinus and in two participants in either the valleculae as well as the piriform sinus. Mean value ± SD of the swallowing score and its subdomains during single task condition and during cognitive dual-task condition with the p value of the post-hoc comparison of the two conditions are shown in Table 1. Mean value ± SD of the swallowing score and its subdomains during single task condition and during motor dual-task condition as well as the p value of the post-hoc comparison are shown in Table 2. 11 participants (41%) presented with a worsening of swallowing function (increase of > 30% in the swallowing score) during the cognitive dual-task compared to baseline. All participants were able to recall the 6-digit number correctly after the cognitive dual-task. The period between presentation of the 6-digit number and recalling it was 90.3 ± 26.3 s. 18 participants (67%) showed a worsening of swallowing function in the motor dual-task. During the motor dual-task the average number of clicks with the right hand was 276 ± 50 and with the left hand 250 ± 41. The duration of white out as defined by Mozzanica et al.^40^ did not differ significantly for any of the consistencies between normal swallowing and either task (Supplementary Table S1).

**Table 1:** Comparison of the swallowing score and its subdomains between single-task and cognitive dual-task conditions.

	Baseline	Dual-task	*p* value
**Swallowing score**	5 ± 3	6 ± 5	0.118
Premature bolus spillage	3 ± 3	4 ± 3	0.222
Penetration/aspiration	0 ± 0	0 ± 0	n.a
Pharyngeal residue	2 ± 3	2 ± 4	0.359

Mean value ± SD of the swallowing score and its subdomains during single task condition and during cognitive dual-task condition with the p value of the post-hoc comparison.

*n.a*.  not applicable.

**Table 2:** Comparison of the swallowing score and its subdomains between single-task and motor dual-task conditions.

	Baseline	Dual-task	*p* value
**Swallowing score**	5 ± 4	8 ± 5	< 0.001*
Premature bolus spillage	3 ± 3	5 ± 3	0.013*
Penetration/aspiration	0 ± 0	0 ± 0	n.a
Pharyngeal residue	2 ± 3	3 ± 4	0.002*

Bold value indicates the *p* < 0.05.

Mean value ± SD of the swallowing score and its subdomains during single task condition and during motor dual-task condition and the p value of the post-hoc comparison.

*n.a*. not applicable.

## Discussion

The main finding of our study is that oropharyngeal swallowing was significantly impaired by concurrent motor dual-task interference while cognitive dual-tasking did not significantly change the swallowing score. Worsening of swallowing function during motor dual-task was driven by increase of pharyngeal residue and premature bolus spillage but not by penetration or aspiration. There are two well established theories that are used to explain dual-task interference. The “capacity sharing theory” postulates that attention demanding mental capacity is a limited resource. If two tasks are performed simultaneously, both tasks share limited mental capacity^41^. This can deteriorate performance in one or both of the concurrent tasks, especially when both tasks require overlapping neural processing^42^. According to the “bottleneck theory”, neural overlapping tasks can only be performed in serial order. This leads to a bottleneck where one task is interrupted while the other is being processed, resulting in a delay^43^. Historically, deglutition was seen as a primary reflexive process that was hypothesized to be automatically controlled and executed by the brainstem^44^. Contrary to this hypothesis, our study suggests that oropharyngeal swallowing requires mental capacity and is thus influenced by concurrent attention demanding tasks. Consistent with this, recent studies have drawn attention to the involvement of several cortical areas both in physiological swallowing as well as in dysphagia rehabilitation^45^. Neuroimaging studies show that the motor cortex and the basal ganglia as extrapyramidal motor network are involved in the central control of swallowing^46–48^. Therefore, swallowing probably shares an overlapping neural network with other motor tasks. This explains why dual-task interference occurs in swallowing similar to other motor processes such as gait.

Only few studies have examined the effect of cognitive dual-task and divided attention on swallowing function. Dodderi et al. investigated the effect of a number recognition task on clinical swallowing parameters. They reported decreased volume and increased time per swallow as sign for less efficient swallowing besides decreased reaction time during the number recognition task^49^. Daniels et al. applied a dual-task paradigm to examine the cortical lateralisation of swallowing^50^. They used a visual-spatial line orientation task that represented right hemispheric and a silent word repetition task that represented left hemispheric cortical processing. The concurrent line orientation task reduced the number of swallows and the language task led to smaller volumes per swallow. The authors therefore conclude that both tasks deteriorate swallowing performance and that swallowing is represented bilaterally. Brodsky et al. reported dual-task interference during the anticipatory phase of swallowing but not for the oropharyngeal phase in a forced choice, reaction time response to auditory stimuli^51^. In their study, the duration of the oropharyngeal phase did not change significantly during dual-task, similar to the findings in the study presented. Troche et al. assessed patients with Parkinson’s disease and dysphagia using videofluoroscopy during a cognitive number repeating task and found a shortening of the pharyngeal transit time^52^. In the overall group, dual-task did not significantly increase the penetration aspiration scale. This is in line with the results of our study in which dual-task did not worsen penetration and aspiration in healthy individuals. However, in the patients with mild cognitive impairments, dual-task led to a worsening of the penetration aspiration scale in their study. This could be a hint, that cognitive deficits might be necessary to result in cognitive dual-task interference and explain why there was no significant worsening of swallowing function in our study. Contradictory, patients with severe cognitive impairment improved in the penetration aspiration scale during cognitive dual-task. Other parameters such as residue or premature bolus spillage that showed dual-task interference in our study were not analysed. In their follow-up study with healthy participants, Brodsky et al. reported dual-task interference in patients with Parkinson’s disease during the anticipatory phase of swallowing, which was reflected in a prolonged reaction time to auditory stimuli in the concurrent dual cognitive task^53^. As opposed to the findings by Troche et al. in patients suffering from Parkinson’s disease, here, the duration of the oropharyngeal transit did not change significantly during dual-task condition. Reynolds et al. reported that patients with Parkinson’s disease drooled more often and swallowed less frequently during a concurrent task in which information had to be memorized^54^. They conclude that drooling during divided attention can be increased due to impaired swallowing.

The fact that there was no significant cognitive dual-task cost in our study might be explained by the study cohort of healthy, mainly young participants with no cognitive impairment. The task to continuously repeat a six-digit number with rather low cognitive load might not have impacted mental capacity. Further, there is evidence that healthy, young participants tend to prioritize motor performance over concurrent cognitive tasks^55^ and cognitive motor interference of the gait is associated with age^36^. These effects may also apply to cognitive interference with swallowing and our mainly young subjects may have prioritized swallowing function over number repeating. Future studies could investigate tasks in participants with cognitive impairment or tasks with higher cognitive load in healthy individuals and also assess the performance in the concurrent cognitive task.

One study also investigated a motor dual-task paradigm on swallowing. Similar to our study, Daniels et al. applied a task which consisted of either left- or right-hand finger tapping^50^. Motor dual-task significantly reduced number of swallows and volume per swallow, whereas the language task only reduced volume per swallow and the line orientation task only reduced number of swallows. In line with the results of our study, the authors suggested that motor dual-task represents more overlapping neural processes in comparison to cognitive dual task and thus results in greater dual task interference. Accordingly, in dual-task interference of the gait, motor tasks also seem to have a greater impact compared to cognitive tasks^56^. However, it must be considered that in most of the dual-task swallowing studies (with exception of Troche et al.) no instrumental diagnostics were used, and therefore besides volume, number of swallows and swallowing timing no differentiated assessment of swallowing function was possible.

In our study, participants in baseline FEES showed minor subclinical signs of premature bolus spillage and pharyngeal residue as similarly described in the literature^57^. Dual-task resulted in subclinical worsening of premature bolus spillage and pharyngeal residue. Premature bolus spillage is associated with a loss of oral bolus control. The oral phase of swallowing is linked to cognitive demanding cortical processing and conversely cognitive deficits primarily result in oral swallowing impairment^58,59^. However, residue in the pharynx indicate pharyngeal swallowing impairment. The results of our study therefore show, that dual-task not only interferes with the oral but also with the pharyngeal phase of swallowing. This could be an indication, that pharyngeal swallowing might require more attention demanding processing and cortical control than previously assumed. The subscale of penetration and aspiration did not worsen under dual-task, indicating that there was no dual-task cost on the most essential safety function of swallowing. Possibly, this domain of swallowing function is indeed controlled more reflexively and rather relies on involuntary aspects e.g. intact protective reflexes^60^ and the integration of pharyngeal sensation^61^.

The results of this study could be clinically relevant in two respects: on the one hand, it could be possible to unmask dysphagia at an early stage of manifestation by dual-task interference. This would be particularly relevant for chronic progressive diseases in which an early behavioural therapy may contribute to maintaining a functional swallowing reserve. Dual-task interference of the gait in patients with Parkinson’s disease is useful to predict future falls^56^. Similarly, dual-task interference of swallowing could be useful to predict the onset of Parkinson's disease related dysphagia. On the other hand, it might be therapeutically useful to recommend patients with worsening of swallowing during dual-task to particularly focus on swallowing while eating and to avoid dual-task situations. To further explore the relevance of dual-task during FEES in a more clinical setting, we intend to perform FEES including dual-task conditions in patients suffering from Parkinson’s disease as well as healthy elderly.

There are important limitations that must be considered. First, the performance of the concurrent cognitive or motor dual-task was not controlled. Particularly in the cognitive dual-task it was therefore not possible to monitor whether the task was carried out continuously during FEES. Also, the cognitive dual-task may have been too simple for young and healthy participants to show larger effects on the swallowing function and a more complex task may be more suitable (e.g. more digits to remember). Second, FEES took significantly longer during the single-task condition compared to either dual-task. We assume that this is to attributed to constant motivation—either to click as fast as possible or to repeat the number in mind digit by digit—during both dual-task conditions. During the single-task condition, participants were only asked to swallow when they wanted which may influence swallowing physiology. Third, since the single task was performed before any of the two dual-task conditions, fatigue may have contributed to a worse swallowing function during the prolonged FEES. In future studies either a randomization of all three conditions and/or defined periods of rest should be considered to compensate for this. Fourth, during the FEES examination, the investigator and the participants were not blinded to the respective condition, which may have affected the outcome. Fifth, the study population consisted of healthy, mainly young volunteers. The results might be different in older subjects of patients with neurological conditions. Further studies in different patient groups are necessary to assess the clinical relevance of the findings.

In summary, our study indicates that a concurrent motor dual-task (but not a cognitive task) influences oropharyngeal swallowing leading to premature bolus spillage and pharyngeal residue in healthy, young participants. This suggests that oropharyngeal swallowing is not exclusively reflexive but requires attention demanding cortical processing. Future studies in different patient populations are needed to investigate the potential diagnostic capability of dual-task FEES in the early detection of dysphagia or the therapeutic benefit of avoiding dual task situations.

## Methods

### Subjects

We recruited 27 healthy individuals to participate in this prospective study. Participants had been only included if no neurological pre-existing conditions or other diseases associated with dysphagia were present.

### FEES-examination

FEES was performed following a stepwise, well established protocol with testing of 3 different food consistencies in the following order: 3 trials of 8 ml of green jelly (semi-solid), 3 trials of 5 ml blue-dyed liquid, and 3 trials of white bread (solid) with a size of approximately 3 cm × 3 cm × 0.5 cm^30^.

### Dual-task paradigm

**Figure 1:**
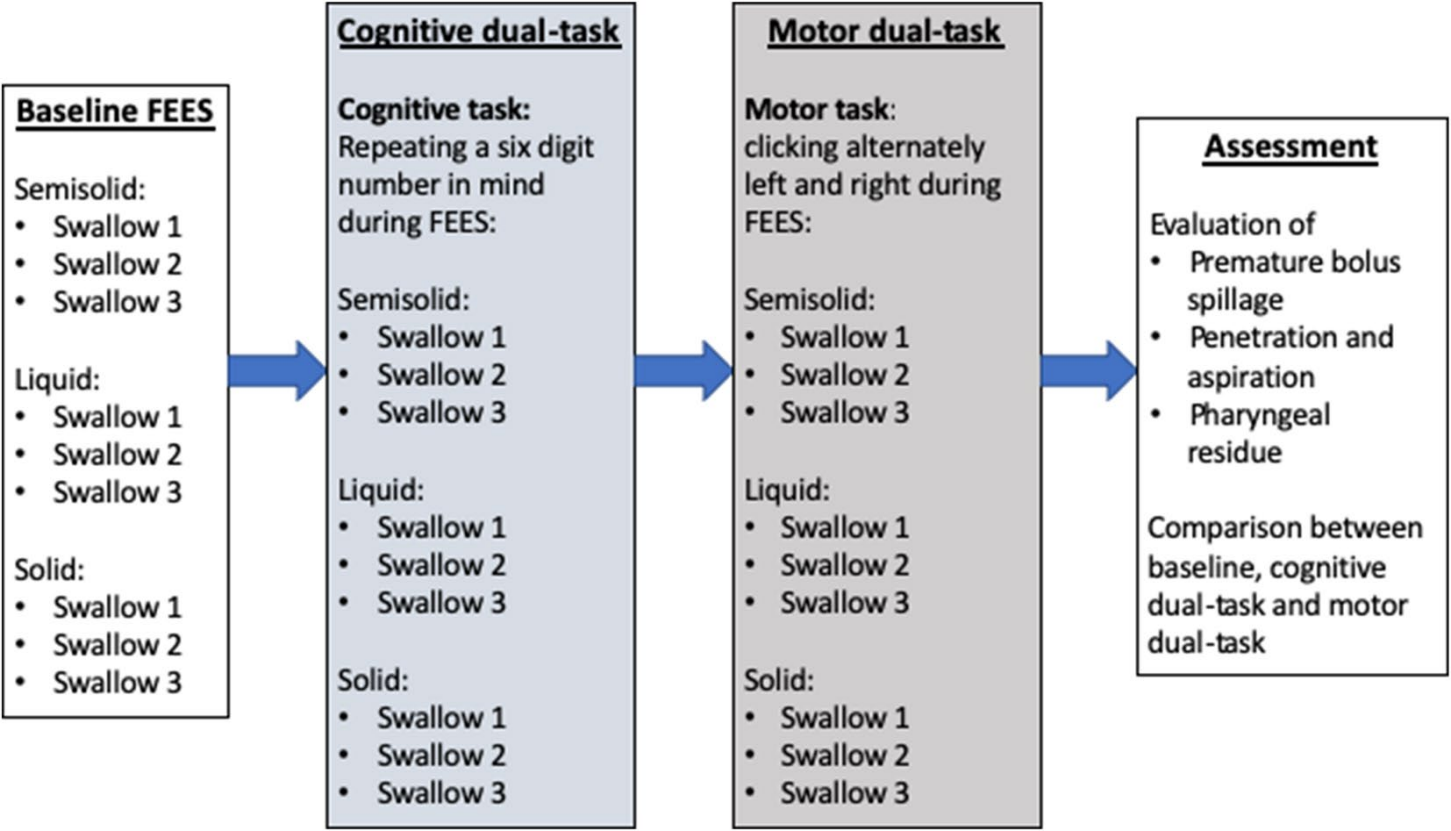
illustration of the study protocol. Cognitive- and motor dual-task were applied in random order.

Three task blocks were carried out: a baseline swallowing task (without interference), and in randomised order a cognitive dual-task**,** and a motor dual-task. In the cognitive task, the participant was given a six-digit number, which had to be repeated aloud correctly three times before beginning with FEES. The participant was then asked to repeat the number continuously in mind digit by digit. During the subsequent swallowing study, the participant was constantly reminded to repeat the digits in mind. At the end of the cognitive dual-task the participant was asked to repeat the number again aloud. In the motor dual-task, the participant received two click devices (AFUNTA, Mini Hand Tally Counter), one in each hand. The click devices were operated by pressing a button. The participant was instructed to click alternately left and right as quickly as possible during the swallowing study and the number of clicks were noted for each hand afterwards. If the speed of clicking decreased, the participant was motivated to increase the speed of clicking. The FEES examination was recorded on video and stored on hard drive for later evaluation. Duration of white out as defined by Mozzanica et al.^40^ was gathered for each swallow. The study protocol is illustrated in Fig. 1.

### Assessment of swallowing function

To assess global swallowing function, we used a score which evaluates three parameters of swallowing function: (1) premature bolus spillage, (2) penetration and aspiration, (3) residue in the pharynx. Each parameter is rated on a scale from 0 (normal) to 4 (severe impairment) for every trial and each food consistency, contributing to an overall cumulative score of a maximum of 108. This score is particularly sensitive to changes in oropharyngeal swallowing function and has recently been validated^62^. This score was originally developed to evaluate Levodopa responsiveness of the swallowing function in patients suffering from Parkinson’s disease, similar to the evaluation of the improvement of motor function during a Levodopa challenge.

### Statistical analysis

Descriptive statistics was applied to quantify demographics and oropharyngeal swallowing function. The data is presented as frequencies for gender distribution and mean values ± standard deviation (SD) for age, the swallowing score und the subdomains of the swallowing score (premature bolus spillage, penetration/aspiration, pharyngeal residue). Since repeated measures ANOVA with a Bonferroni correction is relatively robust to sphericity and normality violations^63^, we used this statistical approach to test whether the swallowing score as well as the subdomains differed between the paradigms, baseline, cognitive dual-task and motor dual-task. If Mauchly’s test indicated that the assumption of sphericity was violated a Greenhouse–Geisser correction was applied. A post-hoc test using the Bonferroni correction was used to compare the tested conditions. In addition, it was determined for each subject whether the cognitive or motor dual-task resulted in a deterioration of swallowing function, which was defined as an increase of the score by > 30% compared to baseline. This cut-off value was chosen according to the original protocol in which a relevant change of dysphagia is defined as a change of > 30% on the score^62^.

### Ethics

Written informed consent was obtained from all participants prior to the study. The study protocol was approved by the local ethics committee (Ethikkommision der Ärztekammer Westfalen-Lippe und Westfälischen Wilhelms-Universität, approval number: 2017-183-f-S). All investigations were performed in accordance with the Declaration of Helsinki and with good clinical practice.

## Supplementary information

Supplementary Table S1.

## Data Availability

The datasets generated during and analysed during the current study are not publicly available due to legal reasons imposed by the University Hospital Muenster but are available from the corresponding author on reasonable request.
