# Supplementary material for: Effects of cognitive and motor dual-tasks on oropharyngeal swallowing assessed with FEES in healthy individuals
Source: Sci Rep. 2020 Nov 23;10:20403. doi: 10.1038/s41598-020-77421-3 (PMC7683567; doi:10.1038/s41598-020-77421-3)
Supplement: Supplementary file 1 — Supplementary Table S1. [file 41598_2020_77421_MOESM1_ESM.docx]

Table 3 Duration of white out during normal swallowing and swallowing during motor and cognitive dual-task for the consistencies semi-solid, liquid and solid (mean value ± standard deviation).

|  |  | **baseline** | **motor**  **dual-task** | **cognitive**  **dual-task** | ***p*-value** |
| --- | --- | --- | --- | --- | --- |
| **semi-solid** | | 0.66 ± 0.11 | 0.64 ± 0.12 | 0.64 ± 0.08 | 0.069^+^ |
| **liquid** |  | 0.62 ± 0.11 | 0.61 ± 0.10 | 0.61 ± 0.09 | 0.312^+^ |
| **solid** |  | 0.72 ± 0.14 | 0.70 ± 0.11 | 0.69 ± 0.12 | 0.299^+^ |

Footnote: ^+^ = Friedman-test
